# Supplementary material for: Human endogenous oxytocin and its neural correlates show adaptive responses to social touch based on recent social context
Source: eLife. 2023 May 9;12:e81197. doi: 10.7554/eLife.81197 (PMC10168694; doi:10.7554/eLife.81197)
Supplement: Supplementary file 1. — All contrasts are thresholded at P<0.002, cluster-size thresholded at alpha = 0.05 FWE for n=27 complete functional datasets. For each cluster under each contrast heading, size in voxels, location, maximum T score, and MNI coordinates (x, y, z) are given. *=region of interest analysis. [file elife-81197-supp1.docx]

**Supplementary Table 1.** Paired T-tests for partner vs stranger during each of 2 functional runs (first, second), modeled with linear mixed effects and weighted by individual change in OT levels as covariates. All contrasts thresholded at *p* < 0.002, cluster-size thresholded at *alpha* = 0.05 FWE for n = 27 complete functional datasets. For each cluster under each contrast heading, size in voxels, location, maximum *T* score, and MNI coordinates (x, y, z) are given. * = region of interest analysis.

**Partner First > Stranger First**

| **Cluster (size)** | **Peaks Locations** | **T (x, y, z)** |
| --- | --- | --- |
| #1 (72) | Left Raphe Nuclei | 5.76 (-11, -47, -41) |
|  | Right Raphe Nuclei | 4.46 (7, -44, -38) |
| #2 (15) | Left Hypothalamus* | 4.16 (-2, -2, -8) |

**Partner Second > Stranger Second**

| **Cluster (size)** | **Peaks Locations** | **T (x, y, z)** |
| --- | --- | --- |
| #1 (223) | Right Angular Gyrus | 5.09 (40, -53, 25) |
|  |  | 4.70 (52, -62, 31) |
|  | Right Supramarginal Gyrus | 4.39 (46, -44, 28) |
|  | Right Superior Temporal Gyrus | 4.02 (52, -44, 16) |
| #2 (166) | Right Middle Temporal Gyrus | 5.30 (46, 4, -29) |
|  |  | 4.77 (55, -2, -29) |
|  | Right Medial Temporal Pole | 5.09 (43, 16, -35) |
| #3 (163) | Right Anterior Cingulate Cortex | 4.74 (13, 49, 10) |
|  | Right Superior Medial Gyrus | 4.61 (7, 67, 1) |
|  | Right Mid Orbital Gyrus | 4.14 (10, 49, -5) |
|  | Left Mid Orbital Gyrus | 4.04 (-8, 55, -2) |
| #4 (88) | Right Superior Frontal Gyrus | 6.07 (25, 19, 64) |
|  |  | 5.66 (19, 28, 61) |
