# Supplementary material for: Human endogenous oxytocin and its neural correlates show adaptive responses to social touch based on recent social context
Source: eLife. 2023 May 9;12:e81197. doi: 10.7554/eLife.81197 (PMC10168694; doi:10.7554/eLife.81197)
Supplement: Supplementary file 2. — All contrasts are thresholded at P<0.002, cluster-size thresholded at alpha = 0.05 FWE for n=18 complete functional datasets. For each cluster under each contrast heading, size, location, maximum T score, and MNI coordinates (x, y, z) are given. [file elife-81197-supp2.docx]

**Supplementary Table 2.** Paired T-test for partner vs stranger in partner first group, modeled with linear mixed effects and weighted by individual mean cortisol levels as covariate. All contrasts thresholded at *p* < 0.002, cluster-size thresholded at *alpha* = 0.05 FWE for n = 18 complete functional datasets. For each cluster under each contrast heading, size, location, maximum *T* score, and MNI coordinates (x, y, z) are given.

**Partner First > Stranger Second**

| **Cluster (size)** | **Peaks Locations** | **T (x, y, z)** |
| --- | --- | --- |
| #1 (495) | Left Anterior Cingulate Cortex | 7.94 (-8, 25, 16) |
|  |  | 7.14 (-2, 34, 16) |
|  |  | 7.08 (-11, 46, 10) |
|  | Right Superior Medial Gyrus | 5.95 (7, 58, 10) |
|  |  | 5.49 (-8, 64, 13) |
|  | Left Mid Orbitofrontal Gyrus | 5.05 (-8, 43, -5) |
|  |  | 4.43 (-8, 52, -11) |
|  | Right Mid Orbitofrontal Gyrus | 4.70 (4, 67, -8) |
| #2 (180) | Right Calcarine Gyrus | 6.11 (25, -74, 7) |
|  |  | 5.54 (10, -74, 10) |
|  |  | 5.42 (-11, -65, 4) |
|  | Right Lingual Gyrus | 5.54 (7, -71, -2) |
|  | Left Calcarine Gyrus | 4.97 (-2, -80, 10) |
| #3 (81) | Left Temporal Pole | 6.32 (-59, 7, -5) |
|  | Left Parietal Operculum | 6.32 (-59, -2, 13) |
