# Supplementary material for: Human endogenous oxytocin and its neural correlates show adaptive responses to social touch based on recent social context
Source: eLife. 2023 May 9;12:e81197. doi: 10.7554/eLife.81197 (PMC10168694; doi:10.7554/eLife.81197)
Supplement: Supplementary file 3. — All contrasts are thresholded at P<0.002, cluster-size thresholded at alpha = 0.05 FWE for N=23 complete functional datasets. For each cluster under each contrast heading, size, location, maximum F score, and MNI coordinates (x, y, z) are given. [file elife-81197-supp3.docx]

**Supplementary Table 3.** Regressor created by linear interpolation of serial OT samples, convolved with canonical HRF and modeled with factors toucher (partner, stranger) and order (first or second encounter), at time points 2 and 2.5 min preceding plasma sample collection. All contrasts thresholded at *p* < 0.002, cluster-size thresholded at *alpha* = 0.05 FWE for N = 23 complete functional datasets. For each cluster under each contrast heading, size, location, maximum *F* score, and MNI coordinates (x, y, z) are given.

**2 minutes preceding sample collection**

**Interaction: Familiarity*Order**

| **Cluster (size)** | **Peaks Locations** | **F (x, y, z)** |
| --- | --- | --- |
| #1 (250) | Left Precuneus | 33.04 (-8, -62, 67) |
|  |  | 17.03 (-14, -50, 79) |
|  | Right Precuneus | 25.65 (10, -65, 58) |
|  |  | 20.84 (16, -44, 49) |
|  |  | 20.70 (13, -50, 58) |
| #2 (87) | Right Postcentral Cingulate Cortex | 25.91 (10, -53, 10) |
|  | Left Precuneus | 24.83 (-5, -53, 13) |
| #3 (63) | Left Inferior Parietal Lobule | 37.21 (-50, -54, 63) |

**2.5 minutes preceding sample collection**

**Interaction: Familiarity*Order**

| **Cluster (size)** | **Peaks Locations** | **F (x, y, z)** |
| --- | --- | --- |
| #1 (234) | Right Precuneus | 27.24 (1, -47, 61) |
|  |  | 26.64 (16, -44, 52) |
|  | Left Precuneus | 21.65 (-2, -65, 55) |
|  |  | 20.62 (-2, -80, 46) |
|  |  | 19.87 (-8, -59, 67) |
|  |  | 16.31 (-8, -47, 55) |
|  | Right Paracentral Lobule | 14.71 (7, -35, 52) |
