# Supplementary material for: Human endogenous oxytocin and its neural correlates show adaptive responses to social touch based on recent social context
Source: eLife. 2023 May 9;12:e81197. doi: 10.7554/eLife.81197 (PMC10168694; doi:10.7554/eLife.81197)
Supplement: Supplementary file 4. — All contrasts are thresholded at P<0.005, cluster-size thresholded at alpha = 0.05 FWE for n=18 complete functional datasets. For each cluster under each contrast heading, size, location, maximum T score, and MNI coordinates (x, y, z) are given. [file elife-81197-supp4.docx]

**Supplementary Table 4.** Paired T-test for partner vs stranger in partner first group, modeled with linear mixed effects and weighted by individual difference in pleasantness ratings as covariate. All contrasts thresholded at *p* < 0.005, cluster-size thresholded at *alpha* = 0.05 FWE for n = 18 complete functional datasets. For each cluster under each contrast heading, size, location, maximum *T* score, and MNI coordinates (x, y, z) are given.

***Partner First > Stranger Second***

| **Cluster (size)** | **Peaks Locations** | **T (x, y, z)** |
| --- | --- | --- |
| #1 (114) | Right Inferior Temporal Gyrus | 4.23 (37, -2, -41) |
