# Supplementary material for: Human endogenous oxytocin and its neural correlates show adaptive responses to social touch based on recent social context
Source: eLife. 2023 May 9;12:e81197. doi: 10.7554/eLife.81197 (PMC10168694; doi:10.7554/eLife.81197)
Supplement: Supplementary file 5. — All contrasts are thresholded at P<0.002, cluster-size thresholded at alpha = 0.05 FWE for n=37 functional datasets. For each cluster under each contrast heading, size in voxels, location, maximum T score, and MNI coordinates (x, y, z) are given. Negative BOLD in boldface. [file elife-81197-supp5.docx]

**Supplementary Table 5.** Conjunction analyses showing common activations for partner and order factors (partner, stranger, first, second). All contrasts thresholded at *p* < 0.002, cluster-size thresholded at *alpha* = 0.05 FWE for n = 37 functional datasets. For each cluster under each contrast heading, size in voxels, location, maximum *T* score, and MNI coordinates (x, y, z) are given. Negative BOLD in boldface.

***Partner First*** ***∩ Stranger Second ∩ Stranger First ∩ Partner Second***

| **Cluster (size)** | **Peaks Locations** | **T (x, y, z)** |
| --- | --- | --- |
| #1 (2264) | Left Supramarginal Gyrus | 11.68 (-56, -23, 43) |
|  |  | 10.47 (-50, -32, 25) |
|  | Left Postcentral Gyrus | 11.18 (-50, -32, 52) |
|  |  | 10.80 (-35, -41, 61) |
|  |  | 10.48 (-41, -35, 67) |
|  | Left Precentral Gyrus | 10.79 (-29, -14, 67) |
|  |  | 7.12 (-59, 7, 31) |
|  | Left Superior Parietal Lobule | 8.99 (-23, -44, 73) |
|  | Left Parietal Operculum | 8.56 (-44, -5, 7) |
|  | Left Supplementary Motor Area | 7.56 (-5, -5, 58) |
| #2 (356) | Right Calcarine Gyrus | 6.11 (25, -74, 7) |
|  |  | 5.54 (10, -74, 10) |
|  |  | 5.42 (-11, -65, 4) |
|  | Right Lingual Gyrus | 5.54 (7, -71, -2) |
|  | Left Calcarine Gyrus | 4.97 (-2, -80, 10) |
| #3 (332) | Right Supramarginal Gyrus | 6.74 (58, -20, 22) |
|  |  | 5.90 (52, -26, 34) |
|  |  | 5.75 (37, -35, 43) |
| #4 (118) | Right Cerebellum | 8.82 (25, -50, -26) |
|  |  | 5.07 (10, -59, -21) |
| #5 (115) | Right Cerebellum | 8.47 (19, -62, -53) |
| #6 (83) | Right Precentral Gyrus | **-5.96** (31, -26, 61) |
|  |  | **-5.48** (37, -20, 52) |
| #7 (78) | Right Supplementary Motor Area | 6.95 (4, 7, 55) |
| #8 (14) | Left Insula | 4.50 (-29, 10, -20) |
| #9 (11) | Left Middle Cingulate Cortex | 5.34 (-8, 13, 43) |
| #10 (9) | Right Amygdala | 4.71 (31, 4, -20) |
| #11 (7) | Right Insula | 4.93 (28, 16, -17) |
| #12 (5) | Right Inferior Frontal Gyrus | 4.96 (25, 13, -20) |

***Partner First ∩ Stranger First***

| **Cluster (size)** | **Peaks Locations** | **T (x, y, z)** |
| --- | --- | --- |
| #1 (2893) | Left Precentral Gyrus | 13.98 (-29, -14, 67) |
|  | Left Supramarginal Gyrus | 12.48 (-59, -23, 43) |
|  |  | 12.00 (-53, -32, 43) |
|  | Left Superior Parietal Lobule | 11.83 (-32, -44, 61) |
|  |  | 10.05 (-26, -47, 73) |
|  | Left Postcentral Gyrus | 11.28 (-41, -35, 70) |
|  |  | 10.45 (-53, -29, 58) |
|  |  | 10.21 (-41, -32, 49) |
|  | Left Parietal Operculum | 9.45 (-44, -5, 7) |
|  | Left Supplementary Motor Area | 8.55 (-5, -5, 58) |
| #2 (803) | Right Precentral Gyrus | 6.70 (55, 7, 34) |
|  | Right Inferior Frontal Gyrus | 6.47 (58, 13, 22) |
|  |  | 6.23 (52, 16, 4) |
|  |  | 5.63 (34, 4, 28) |
|  | Right Insula | 6.24 (40, 25, 1) |
|  |  | 5.79 (34, 10, -11) |
|  |  | 5.48 (40, 1, 10) |
|  | Right Temporal Pole | 5.44 (31, 16, -26) |
|  | Right Parahippocampal Gyrus | 5.00 (22, 1, -23) |
| #3 (411) | Right Supramarginal Gyrus | 7.00 (67, -26, 19) |
|  |  | 5.91 (37, -35, 43) |
|  |  | 5.73 (49, -26, 34) |
|  | Right Parietal Operculum | 7.00 (55, -20, 22) |
|  | Right Postcentral Gyrus | 5.90 (61, -17, 43) |
|  | Right Inferior Parietal Lobule | 5.44 (37, -41, 55) |
| #4 (264) | Right Cerebellum | 9.78 (28, -56, -35) |
|  |  | 4.93 (10, -59, -11) |
| #5 (213) | Right Cerebellum | 9.69 (22, -56, -53) |
|  |  | 8.84 (16, -68, -50) |
| #6 (176) | Left Cerebellum | 6.66 (-23, -71, -53) |
|  |  | 6.60 (-32, -62, -56) |
| #7 (113) | Right Postcentral Gyrus | **-5.82** (28, -26, 61) |
|  | Right Precentral Gyrus | **-5.55** (34, -20, 49) |
| #8 (35) | Right Caudate | 4.38 (16, 13, 10) |
| #9 (28) | Left Temporal Pole | 4.90 (-29, 7, -20) |
| #10 (14) | Right Caudate Nucleus | 4.83 (7, 1, -5) |
|  |  |  |
|  |  |  |
|  |  |  |

***Partner Second ∩ Stranger Second***

| **Cluster (size)** | **Peaks Locations** | **T (x, y, z)** |
| --- | --- | --- |
| #1 (3121) | Left Postcentral Gyrus | 12.58 (-50, -32, 52) |
|  |  | 11.26 (-38, -32, 46) |
|  |  | 8.11 (-23, -44, 76) |
|  | Left Supramarginal Gyrus | 11.04 (-56, -23, 43) |
|  |  | 9.55 (-50, -35, 25) |
|  |  | 9.23 (-44, -26, 25) |
|  |  | 9.17 (-56, -26, 19) |
|  |  | 8.29 (-71, -29, 25) |
|  | Left Precentral Gyrus | 8.11 (-32, -14, 61) |
|  | Left Parietal Operculum | 7.94 (-44, -5, 10) |
| #2 (615) | Right Insula | 6.25 (43, -2, 1) |
|  |  | 5.48 (25, 16, -17) |
|  |  | 5.46 (40, 25, -5) |
|  |  | 5.04 (40, 1, 16) |
|  | Right Inferior Frontal Gyrus | 6.20 (46, 10, 19) |
|  |  | 6.07 (55, 10, 28) |
|  |  | 5.31 (58, 13, 7) |
|  |  | 5.18 (52, 13, -2) |
|  | Right Amygdala | 5.29 (31, 4, -20) |
| #3 (460) | Right Supramarginal Gyrus | 7.07 (58, -32, 25) |
|  |  | 6.51 (58, -20, 22) |
|  |  | 6.14 (52, -26, 34) |
|  | Right Postcentral Gyrus | 6.05 (34, -38, 49) |
| #4 (138) | Right Cerebellum | 8.22 (25, -50, 29) |
| #5 (132) | Right Cerebellum | 8.00 (16, -65, -53) |
| #6 (86) | Right Precentral Gyrus | **-6.17** (31, -26, 61) |
| #7 (38) | Left Caudate | 5.01 (-11, 13, -2) |
|  |  | 4.68 (-20, 22, 4) |
| #8 (4) | Left Putamen | 4.57 (-17, 10, -2) |
|  |  |  |
|  |  |  |
|  |  |  |

***Partner First ∩ Stranger Second***

| **Cluster (size)** | **Peaks Locations** | **T (x, y, z)** |
| --- | --- | --- |
| #1 (4187) | Left Precentral Gyrus | 16.16 (-29, -14, 67) |
|  | Left Supramarginal Gyrus | 15.80 (-56, -26, 43) |
|  |  | 12.85 (-50, -35, 25) |
|  | Left Postcentral Gyrus | 14.83 (-41, -35, 67) |
|  |  | 14.68 (-38, -32, 46) |
|  | Left Parietal Operculum | 11.46 (-44, -5, 7) |
|  | Left Superior Parietal Lobule | 11.41 (-26, -47, 73) |
|  | Left Supplementary Motor Area | 9.82 (-5, -5, 58) |
|  |  | 9.55 (-14, 1, 67) |
|  |  | 9.17 (4, 7, 55) |
| #2 (2350) | Right Superior Temporal Gyrus | 8.22 (58, -35, 22) |
|  | Right Parietal Operculum | 7.83 (55, -23, 22) |
|  | Right Supramarginal Gyrus | 7.31 (34, -38, 46) |
|  |  | 6.50 (52, -26, 34) |
|  | Right Precentral Gyrus | 7.29 (52, 7, 37) |
|  | Right Superior Parietal Lobule | 7.13 (34, -47, 58) |
|  | Right Insula | 6.89 (34, 10, -11) |
|  |  | 6.68 (40, 1, 16) |
|  | Right Inferior Frontal Gyrus | 6.87 (46, 10, 19) |
|  | Right Superior Frontal Gyrus | 6.49 (34, -8, 58) |
| #3 (417) | Right Cerebellum | 10.60 (25, -50, 26) |
|  |  | 6.25 (10, -59, -11) |
|  | Right Fusiform Gyrus | 5.22 (43, -41, -17) |
| #4 (312) | Right Cerebellum | 10.62 (16, -65, -53) |
| #5 (215) | Right Middle Temporal Gyrus | 6.58 (52, -62, 1) |
| #6 (208) | Left Cerebellum | 7.81 (-23, -74, -56) |
| #7 (193) | Left Middle Temporal Gyrus | 8.66 (-56, -77, 4) |
|  |  | 7.71 (-44, -62, 7) |
| #8 (108) | Right Postcentral Gyrus | **-6.35** (28, -26, 58) |
|  | Right Precentral Gyrus | **-5.36** (34, -20, 49) |
| #9 (73) | Left Cerebellum | 5.05 (-26, -65, -26) |
| #10 (47) | Left Insula | 5.26 (-32, 10, -17) |
|  |  |  |

***Stranger First ∩ Partner Second***

| **Cluster (size)** | **Peaks Locations** | **T (x, y, z)** |
| --- | --- | --- |
| #1 (2460) | Left Postcentral Gyrus | 10.41 (-50, -32, 58) |
|  |  | 7.61 (-35, -32, 73) |
|  |  | 7.27 (-23, -44, 76) |
|  | Left Rolandic Operculum | 9.55 (-47, -23, 22) |
|  | Left Supramarginal Gyrus | 9.12 (-53, -32, 25) |
|  |  | 8.93 (-59, -23, 43) |
|  | Left Superior Parietal Lobule | 8.88 (-32, -44, 61) |
|  | Left Parietal Operculum | 7.35 (-53, -2, 7) |
|  | Left Supplementary Motor Area | 6.90 (-11, -5, 55) |
| #2 (387) | Right Insula | 6.22 (40, 19, -5) |
|  |  | 5.68 (43, 1, -2) |
|  | Right Inferior Frontal Gyrus | 5.64 (55, 16, 7) |
|  |  | 5.29 (58, 13, 25) |
| #3 (332) | Right Supramarginal Gyrus | 6.51 (61, -29, 28) |
|  |  | 5.89 (58, -17, 22) |
|  |  | 5.30 (52, -26, 34) |
|  | Right Inferior Parietal Lobule | 4.65 (40, -35, 46) |
| #4 (147) | Right Precentral Gyrus | **-6.08** (34, -26, 61) |
|  |  | **-5.81** (37, -20, 52) |
| #5 (122) | Right Cerebellum | 7.07 (25, -50, 26) |
|  |  | 4.35 (10, -56, -14 |
| #6 (121) | Right Cerebellum | 6.77 (22, -59, -53) |
| #7 (81) | Right Supplementary Motor Area | 5.05 (7, 13, 55) |
| #8 (29) | Right Caudate | 5.73 (7, 4 -2) |
| #9 (29) | Left Caudate | 4.74 (-8, 16, -2) |
|  |  | 4.40 (-17, 22, 4) |
| #10 (23) | Right Anterior Cingulate Cortex | 4.36 (4, 19, 25) |
|  | Right Middle Cingulate Cortex | 3.96 (4, 25, 34) |
| #11 (19) | Right Insula | 4.55 (28, 22, -17) |
| #12 (14) | Right Amygdala | 4.91 (22, 1, -20) |
|  |  | 4.42 (34, 1, -23) |
| #13 (11) | Left Middle Cingulate Cortex | 4.06 (-5, 16, 37) |
| #14 (6) | Right Caudate | 4.06 (10, 16, 1) |
| #15 (2) | Right Caudate | 5.17 (7, 1, -5) |
|  |  |  |

***Partner First ∩ Partner Second***

| **Cluster (size)** | **Peaks Locations** | **T (x, y, z)** |
| --- | --- | --- |
| #1 (3029) | Left Precentral Gyrus | 13.90 (-29, -14, 67) |
|  | Left Postcentral Gyrus | 11.66 (-32, -41, 61) |
|  |  | 11.11 (-41, -35, 70) |
|  |  | 10.42 (-53, -32, 61) |
|  |  | 10.35 (-41, -32, 49) |
|  | Left Supramarginal Gyrus | 11.44 (-56, -26, 43) |
|  | Left Parietal Operculum | 11.09 (-47, -29, 19) |
|  | Left Superior Parietal Lobule | 9.93 (-23, -44, 73) |
|  | Left Insula | 9.85 (-44, -2, 7) |
|  | Right Supplementary Motor Area | 8.95 (4, 7, 55) |
| #2 (500) | Right Inferior Frontal Gyrus | 6.52 (58, 13, 22) |
|  |  | 6.07 (61, 10, 10) |
|  |  | 5.63 (40, 31, 1) |
|  |  | 5.22 (46, 16, 1) |
|  | Right Insula | 6.03 (37, 1, 16) |
|  |  | 5.62 (43, 1, 1) |
| #3 (467) | Right Parietal Operculum | 7.16 (55, -20, 19) |
|  | Right Supramarginal Gyrus | 5.67 (37, -35, 46) |
| #4 (136) | Right Cerebellum | 9.15 (22, -59, -50) |
|  |  | 8.07 (13, -68, -50) |
| #5 (134) | Right Cerebellum | 8.58 (25, -53, -26) |
|  |  | 5.12 (10, -59, -11) |
| #6 (105) | Right Precentral Gyrus | **-6.20** (28, -23, 58) |
|  |  | **-5.90** (34, -20, 49) |
| #7 (39) | Right Caudate | 4.89 (10, 10, -2) |
|  |  | 4.19 (19, 22, 4) |
| #8 (34) | Right Amygdala | 4.98 (25, 10, -17) |
| #9 (16) | Right Caudate | 5.33 (7, 4, -2) |
| #10 (2) | Right Insula | 4.22 (40, -14, 10) |

***Stranger First ∩ Stranger Second***

| **Cluster (size)** | **Peaks Locations** | **T (x, y, z)** |
| --- | --- | --- |
| #1 (3270) | Left Postcentral Gyrus | 13.01 (-50, -32, 52) |
|  | Left Supramarginal Gyrus | 11.97 (-56, -23, 43) |
|  | Left Postcentral Gyrus | 11.27 (-38, -32, 46) |
|  | Left Supramarginal Gyrus | 10.87 (-53, -32, 25) |
|  | Left Postcentral Gyrus | 10.08 (-38, -41, 61) |
|  | Left Postcentral Gyrus | 8.46 (-23, -44, 76) |
|  | Left Precentral Gyrus | 8.41 (-59, 7, 31) |
|  | Left Precentral Gyrus | 8.20 (-32, -14, 61) |
|  | Left Parietal Operculum | 7.50 (-47, -5, 10) |
|  | Left Parietal Operculum | 6.96 (-56, 4, 13) |
| #2 (952) | Right Precentral Gyrus | 7.28 (52, 7, 37) |
|  | Right Precentral Gyrus | 6.87 (43, 4, 31) |
|  | Right Inferior Frontal Gyrus | 6.38 (52, 19, 7) |
|  | Right Amygdala/Hippocampus | 5.95 (16, -8, 14) |
|  | Right Insula | 5.82 (34, 7, -14) |
|  | Right Putamen | 5.61 (28, 25, 1) |
| #3 (411) | Right Supramarginal Gyrus | 6.72 (58, -32, 25) |
|  | Right Supramarginal Gyrus | 6.56 (61, -17, 25) |
|  | Right Postcentral Gyrus | 6.26 (37, -38, 52) |
|  | Right Postcentral Gyrus | 5.91 (58, -17, 43) |
|  | Right Supramarginal Gyrus | 5.69 (52, -26, 34) |
| #4 (247) | Right Cerebellum | 9.52 (25, -50, -26) |
|  | Right Cerebellum | 5.05 (7, -62, -11) |
| #5 (202) | Right Cerebellum | 8.82 (16, -65, -53) |
| #6 (135) | Left Cerebellum | 6.73 (-26, -68, -53) |
| #7 (92) | Right Postcentral Gyrus | **-5.91** (28, -26, 61) |
| #8 (5) | Left Caudate | 4.15 (-17, 19, 4) |
| #9 (3) | Left Caudate | 4.14 (-11, 16, -2) |
| #10 (3) | Left Caudate | 4.57 (-20, 22, 4) |
| #11 (2) | Left Temporal Pole | 4.10 (-47, 13, -14) |
