# Supplementary material for: Human endogenous oxytocin and its neural correlates show adaptive responses to social touch based on recent social context
Source: eLife. 2023 May 9;12:e81197. doi: 10.7554/eLife.81197 (PMC10168694; doi:10.7554/eLife.81197)
Supplement: Supplementary file 6. — OT = Oxytocin, CORT = Cortisol, LME = Linear Mixed Effects Model (neuroimaging), Regr.=Regressor (neuroimaging). [file elife-81197-supp6.docx]

**Supplementary File 6**. Participants included in each analysis based on analyzable data. OT = Oxytocin, CORT = Cortisol, LME = Linear Mixed Effects Model (neuroimaging), Regr. = Regressor (neuroimaging).

* = Participants excluded from the from Hypothalamus ROI analysis for signal dropout in that region.

P = only the Partner run is included in this analysis.

S = only the Stranger run is included in this analysis.
